# Survival of a Long‐Lived Avian Scavenger: Implications of Age, Season, and Landscape Composition for Mortality Risk

**DOI:** 10.1002/ece3.73226

**Published:** 2026-03-08

**Authors:** Spencer B. Hudson, Eric A. Tillman, Marian L. Wahl, Patrick A. Zollner, Caryn D. Ross, Travis L. DeVault, James C. Beasley, Adrián Naveda‐Rodríguez, Scott A. Rush, Noah M. Osterhoudt, Jeffrey F. Kelly, Adam E. Duerr, Lee A. Humberg, Brett G. Dunlap, Chad Neil, John T. Forbes, Harris Glass, Travis L. Guerrant, Robert W. Byrd, Philip W. Kavouriaris, Andrea K. Darracq, Matthew T. Springer, Bryan M. Kluever

**Affiliations:** ^1^ USDA APHIS Wildlife Services National Wildlife Research Center Gainesville Florida USA; ^2^ Department of Forestry and Natural Resources Purdue University West Lafayette Indiana USA; ^3^ Savannah River Ecology Laboratory, Warnell School of Forestry and Natural Resources University of Georgia Athens Georgia USA; ^4^ Department of Wildlife, Fisheries and Aquaculture Mississippi State University Starkville Mississippi USA; ^5^ School of Biological Sciences University of Oklahoma Norman Oklahoma USA; ^6^ Conservation Science Global, Inc. Cape May New Jersey USA; ^7^ USDA APHIS Wildlife Services Indiana State Program West Lafayette Indiana USA; ^8^ USDA APHIS Wildlife Services West Virginia State Program Elkins West Virginia USA; ^9^ USDA APHIS Wildlife Services Pennsylvania State Program Harrisburg Pennsylvania USA; ^10^ USDA APHIS Wildlife Services Missouri State Program Columbia Missouri USA; ^11^ USDA APHIS Wildlife Services Arkansas State Program Little Rock Arkansas USA; ^12^ Department of Biological Sciences Murray State University Murray Kentucky USA; ^13^ Department of Forestry and Natural Resources University of Kentucky Lexington Kentucky USA

**Keywords:** black vulture, demography, ecology, mark‐encounter, satellite telemetry

## Abstract

Despite the ecological importance of avian scavengers such as vultures, demographic information that is essential to their conservation and management remains limited. The goal of this study was to evaluate survival and mortality risk in black vultures ( *Coragyps atratus* ), a protected native species of conflict management concern in the United States. Here, we combined monitoring data from a 28‐year period to estimate annual survival rates among age classes and test for seasonal and age‐related patterns in mortality risk. Using dead recovery information, we also summarized the causes and timing of annual mortalities. Additionally, we tested whether mortality risk was affected by aspects of landscape composition and configuration, as well as human development. Average annual survival was high overall (0.95, 95% CI: 0.92–0.98), with estimate precision markedly improved by combining datasets (72.1%–84.2% increase). Mortality risk differed by season and age class such that vultures experienced 68.7% more hazard during the breeding season, and adults experienced 66.2% less hazard than juveniles. Among the mortality causes, 67% were anthropogenic, 4% were natural, and the remaining 29% were unknown. Additionally, greater land cover diversity (Shannon diversity index) reduced mortality risk, whereas measures of landscape configuration and human development had no effect. High survival rates help explain this species' population growth and range expansion and further inform allowable take for sustainable management practices. Moreover, the identified seasonal and age‐related vulnerabilities may help guide lethal control of human–vulture conflicts in an ecologically relevant manner. Maintaining diverse landscapes may also enhance survival overall, facilitating conservation of this species and other avian scavengers.

## Graphical abstract

The goal of this study was to evaluate survival and mortality risk in black vultures ( *Coragyps atratus* ), a protected native species of conflict management concern in the United States. High survival rates from this study help explain this species' population growth and range expansion and further inform allowable take for sustainable management practices.

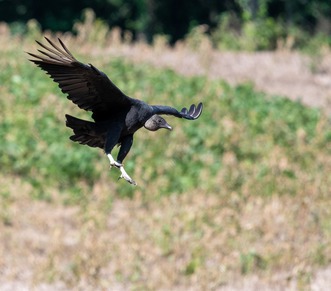

## 1 Introduction

Survival is a critical demographic parameter for wildlife conservation, ultimately affecting individual fitness and population persistence (Sibly and Hone 2002). For avian species with long life expectancies, delayed sexual maturity, and low fecundity (i.e., *K*‐selected; Reznick et al. 2002), population trajectories can be particularly sensitive to ecological drivers affecting survival (Sæther and Bakke 2000; Sergio et al. 2011). Identifying these drivers in addition to measuring survival rates is therefore essential for developing conservation and management practices that mitigate population decline and potential human conflicts (Conover and Conover 2022). However, obtaining reliable estimates of survival can be challenging, as long‐term monitoring programs with extensive field efforts are required for traditional population analyses (e.g., mark‐resight‐recovery designs; White and Burnham 1999). Moreover, different selective pressures and ecological constraints often lead to greater complexity in avian survival rates among geographic areas (Evans et al. 2015; Scholer et al. 2020), demographic groups (Mallord et al. 2024), and phenological events (Robinson et al. 2020). Consequently, in‐depth assessments across these contexts have only been recently reported for a handful of *K*‐selected species (e.g., Naveda‐Rodríguez et al. 2023; Gómez‐López et al. 2025), while several others remain largely understudied around the globe.

Of the avian guilds of concern, obligate scavengers such as vultures provide invaluable ecosystem services through their use of carrion as a food source for survival (e.g., stabilizing food webs; DeVault et al. 2016; McClure and Rolek 2020). As obligate scavengers, vultures have evolved soaring capabilities for energy‐efficient locomotion, enhanced olfactory and visual capacities for carrion detection, and communal roosting behaviors for information sharing, which altogether enable greater foraging success across large search areas (Buckley 1996; DeVault et al. 2003). Due to these adaptations, however, vultures are increasingly found utilizing anthropogenic landscapes that give rise to conflicts with humans (Kluever et al. 2020). Indeed, human‐vulture conflicts have exposed several species to additional mortality factors (e.g., poisoning, collisions, electrocutions, shootings) that further reduce population sustainability (McClure et al. 2018; Hill et al. 2019; Ives et al. 2022).

**FIGURE 1:**
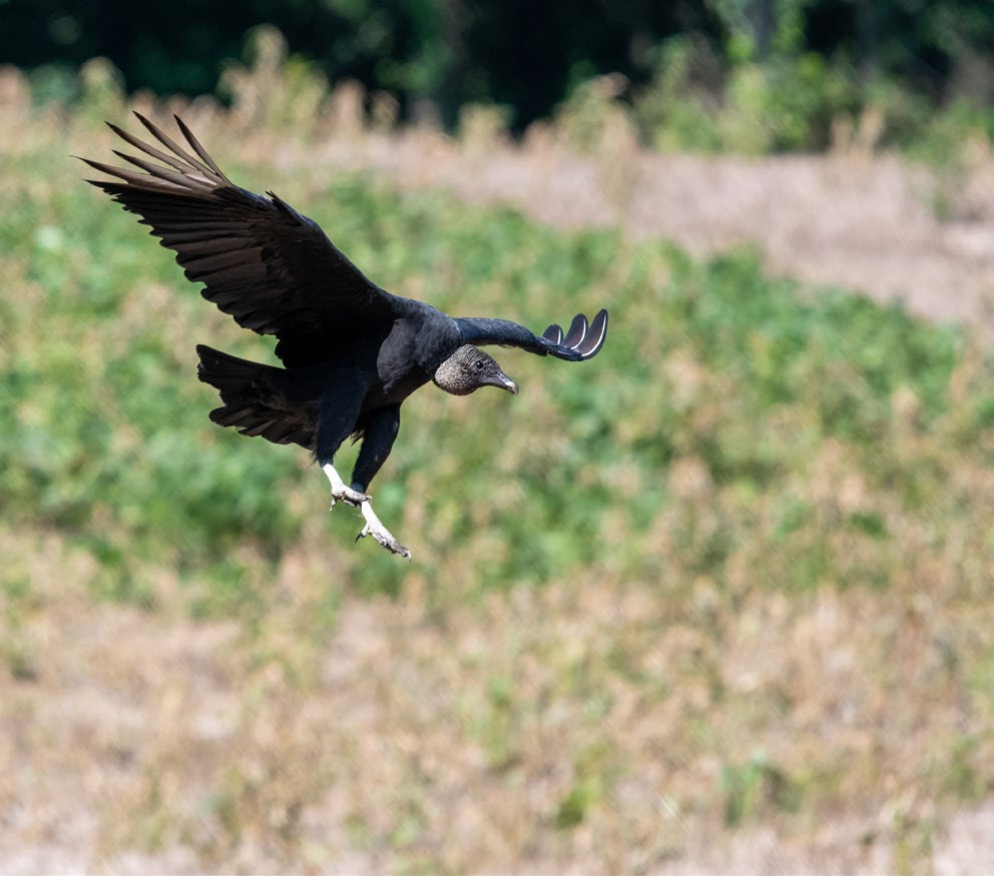
A black vulture ( *Coragyps atratus* ) landing to feed on carrion in Indiana, United States. Photo credit: Dr. Betsy Evans.

Despite reports corroborating vulture populations declining worldwide (Ogada et al. 2012; Callaghan et al. 2021), black vultures ( *Coragyps atratus* ; Figure 1) have instead increased in abundance and expanded their range northward over the past several decades (Avery 2004; Rushing et al. 2020; McClure et al. 2022). Between 2.7 and 6.7 million black vultures have been estimated to be in the United States since 2015, with an intrinsic growth rate of approximately 11% each year (Runge et al. 2009; Zimmerman et al. 2019). Local abundance often varies based on expansion history, whereby greater numbers of black vultures reside in southern versus northern states (e.g., 1,149,817 in Florida and 5291 in Delaware; Zimmerman et al. 2019). The growing prevalence of this species, especially at the current northern range limits, is leading to more frequent interactions and reports of conflict with humans (Kluever et al. 2020). Such conflicts often involve property damage (e.g., roofing materials, vehicle components; Avery and Lowney 2016), perceived livestock depredations (Quinby et al. 2022; Wahl et al. 2023, 2024), or human safety risks (e.g., aircraft collisions, fecal matter exposure; DeVault et al. 2005; Blackwell and Wright 2006; Avery et al. 2011). Notwithstanding the ecological role that black vultures fulfill (DeVault et al. 2016), the extent of their ongoing conflicts has warranted population management.

For black vultures managed in the United States, a variety of methods are employed by federal and state wildlife agencies to mitigate their conflicts in a non‐lethal manner, including but not limited to habitat modification (e.g., removal of roost trees or food sources), exclusion (e.g., tightly spaced spikes or electric tracks on perches), scare devices (e.g., effigies, lasers, inflatable deterrents, or pyrotechnics), and translocation via trapping (Avery and Lowney 2016). However, non‐lethal techniques are not always effective in areas where long‐standing and/or intense conflict has been documented to occur (Kluever et al. 2020, 2024; Wahl et al. 2023). To help reinforce non‐lethal control measures under such circumstances, the U.S. Fish and Wildlife Service (USFWS) can issue permits under the Migratory Bird Treaty Act (16 USC § 703–712) that authorize lethal take of undeterred black vultures when acting in accordance with local laws and city ordinances (50 CFR § 21.41). Importantly, legal requirements regarding the geographic or spatial scale of lethal takes have not been specified, allowing each administrative region of USFWS to allocate depredation permits among federal and state agencies as well as individual stakeholders where applicable. Here, the extent to which black vultures are subject to lethal removals is liable to depend on local abundance, conflict frequency, severity of damages or risks incurred, and the best available science. Yet, given the mortality factors that vultures are already exposed to throughout their range (McClure et al. 2018; Hill et al. 2019), wildlife managers consider lethal control the last recourse, for use when all other options have been exhausted. Of concern here is that lethal removals could be implemented excessively, such that population viability becomes threatened and local extirpation occurs. Determining biologically sustainable levels of lethal take is thus critical in balancing conflict mitigation with population conservation of a protected native species.

Current allowable take estimates for black vultures are based on a prescribed take level framework, which uses demographic rates (e.g., recruitment, survival) to calculate a maximum annual growth rate (Runge et al. 2009; Zimmerman et al. 2019). Despite the importance of these parameters, demographic studies of black vultures have so far been geographically limited to single states, with survival rates derived from inferences of mortality (e.g., nest site turnover) rather than contemporary methods (Rabenold 1986; Rabenold and Decker 1990; Blackwell et al. 2007). To overcome these limitations, previous allowable take models have relied on survival estimates from griffon vultures ( *Gyps fulvus* ; Sarrazin et al. 1994), an unrelated surrogate species that differs ecologically (Runge et al. 2009; Zimmerman et al. 2019). Without robust information on black vulture survival, including estimates for distinct age classes (e.g., subadult versus adult), there is a risk of uncertainty associated with their current allowable take levels. Evaluating survival from multiple data sources that represent different demographic groups, geographic areas, phenological periods, and environmental conditions should altogether inform how lethal management can be both effective and sustainable (Rush et al. 2023).

For this study, we combined monitoring data from a 28‐year period to improve annual survival estimates of black vultures in the United States, and for the first time, investigated seasonal and age‐related patterns in their mortality risk. To evaluate the utility of supplementing satellite telemetry data with incidentally collected mark‐encounter data, we compared survival estimates and their precision as indicators of potential bias. Additionally, we determined whether survival was shaped by environmental conditions, including aspects of human development, landscape composition, and/or landscape configuration. We hypothesized survival rates to be comparable to other large‐sized raptors, and mortality risk to vary with age class and environmental conditions, but not by season. As a *K*‐selected species, black vultures were expected to have high survival rates overall (> 0.9; Newton et al. 2016), but younger vultures were predicted to be at greater risk while overcoming growth and development (Gómez‐López et al. 2025). As a generally sedentary species, we did not expect increased risk following the breeding season that returning migratory vultures experience (e.g., Naveda‐Rodríguez et al. 2023). With greater human development, we expected mortality risk to either decrease from additional food access (Novaes and Cintra 2013, 2015) or increase from exposure to additional mortality factors (Arrondo et al. 2020). Since heterogenous habitats are preferred by black vultures (Hill et al. 2021; Evans et al. 2024), we expected higher survival in landscapes with greater land cover richness, diversity and edge density, and less aggregation and interspersion.

## 2 Materials and Methods

### 2.1 Study Area

**FIGURE 2:**
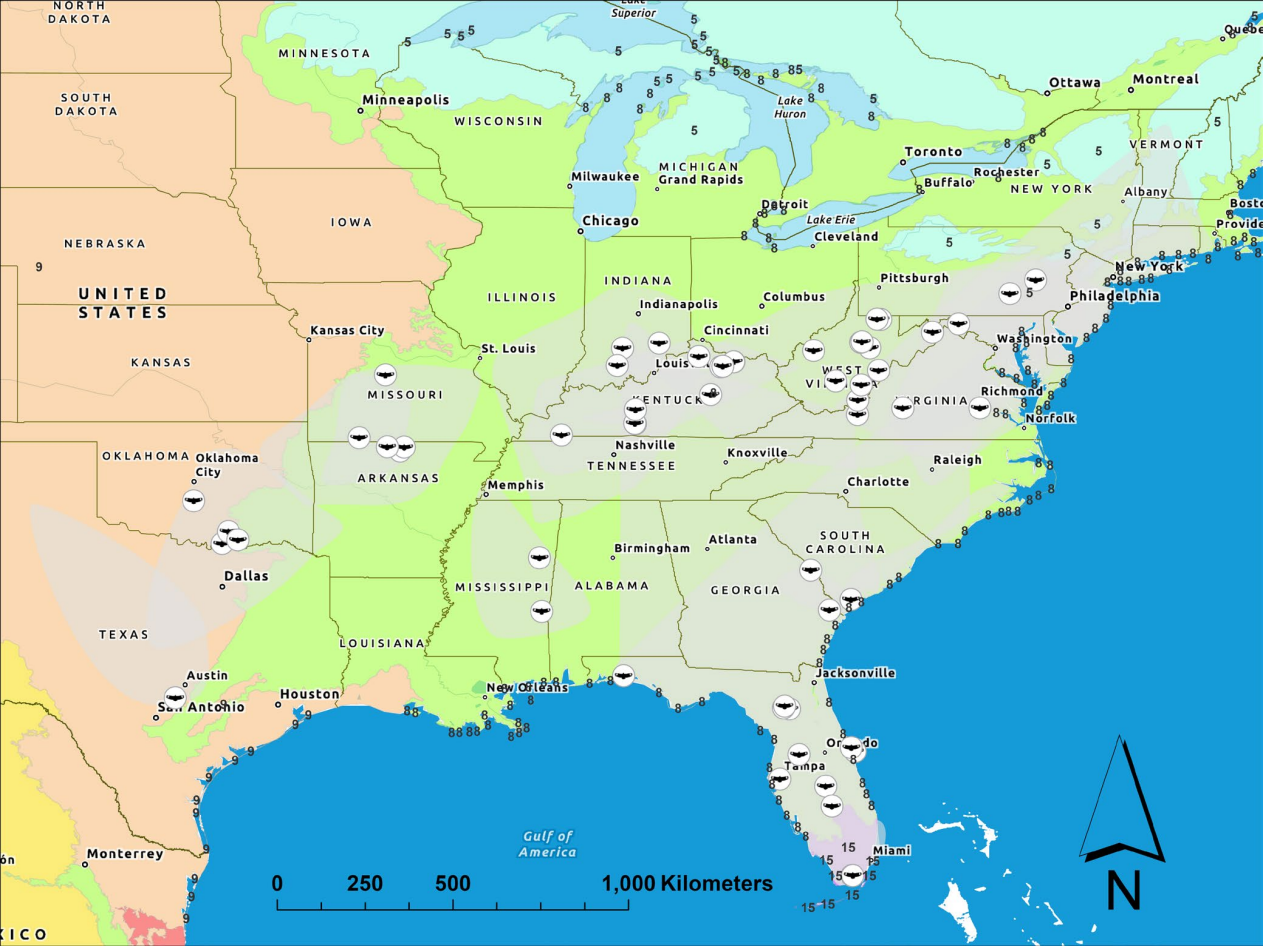
Study area for black vultures in the United States, 1997–2025. Points indicate trapping locations while gray areas represent observed space use of all vultures during the study period. Additional colors and corresponding numbers represent level I ecoregions, including: Tropical Wet Forests (Purple—15), Eastern Temperate Forests (Light Green—8), Great Plains (Light Orange—9), and Northern Forests (Light Blue—5).

The contiguous United States is biogeographically diverse, including twelve level I ecoregions of the Nearctic and Neotropical realms (Omernik and Griffith 2014). Historically, black vultures occupied four of these ecoregions, including the Tropical Wet Forests, Eastern Temperate Forests, Great Plains, and North American Deserts (Buckley et al. 2022). The numerical and spatial expansion of this species over the past several decades has led to a distribution that now encompasses seven of the ecoregions, including the Tropical Wet Forests, Great Plains, Eastern Temperate Forests, North American Deserts, Northern Forests, Temperate Sierras, and Southern Semi‐arid Highlands (Sauer et al. 2017). The geographic extent of this study spanned four of the ecoregions currently inhabited by black vultures, which included 26 states in the southeastern, southwestern, northeastern, and midwestern United States (Figure 2). Monitoring data incorporated herein revealed overlap in space use among vultures from multiple states, corroborating previous findings of a high degree of movement connectivity across the species range (see Zimmerman et al. 2019). Therefore, black vulture survival was assessed at a scale that treated all study birds as a single population.

### 2.2 Field Capture and Processing

Between August 1997 and February 2025, we caught black vultures (*n* = 3281) using baited walk‐in traps, net cannons, and hand nets. Captured vultures were assigned age codes based on information collected at the time of marking (e.g., bill color, amount of feathering and rugosity on the head; Buckley et al. 2022). Age codes followed those used by the U.S. Geological Survey (USGS) Bird Banding Laboratory, including (1) Hatch Year (HY) birds that hatched during the calendar year in which they were marked; (2) After Hatch Year (AHY) birds that hatched before the calendar year of marking, but year of hatch otherwise unknown; and (3) After Second Year (ASY) birds that hatched earlier than the calendar year preceding the marking year, but year of hatch otherwise unknown. Age codes were assigned conservatively, such that only the oldest adults (i.e., those with heavily wrinkled neck skin) were ASY. A unique identifier, based on alphanumeric code and color, was then assigned to each vulture by affixing a tag to their right wing patagium, approximately 3 cm anterior to the humerus to radius‐ulna articulation (Sweeney et al. 1985). Depending on the study, we used either Allflex livestock tags or an alternative makeshift design consisting of vinyl materials fastened with Allflex buttons (Allflex Inc., Dallas, TX, USA).

### 2.3 Transmitter Deployment

Between September 2006 and February 2025, we equipped a subset of the 3281 wing‐tagged vultures with satellite transmitters (*n* = 184) as authorized by U.S. Federal Bird Banding Permits where applicable. As detailed in Humphrey and Avery (2014), we affixed each transmitter using a backpack harness threaded with Teflon ribbons (DuPont Inc., Wilmington, DE, USA) and fastened with a Fastex snap rivet (Illinois Tool Works, Glenview, IL, USA). Deployed units included solar‐powered ARGOS‐PTT GPS transmitters and solar‐powered GPS/GSM transmitters (Microwave Telemetry, Columbia, MD, USA; Ornitela, Vilnius, LT; Northstar Science and Technology, King George, VA, USA; Cellular Tracking Technologies, Rio Grande, NJ, USA). Transmitter weights (range = 30–70 g) adhered to body mass limits permitted by the USGS Bird Banding Laboratory. Transmission duty cycles depended on satellite transmitter capabilities and research needs of each project, resulting in variable sampling intervals overall (every 1–1500 min).

### 2.4 Transmitter and Mark‐Encounter Data

Transmitter‐equipped vultures (hereafter “transmitter vultures”) were continuously monitored by government and academic researchers for location and mortality status throughout their respective study periods. Differing research priorities and resources available (e.g., funding, personnel) for each source of monitoring data prohibited a standardized approach to carcass and/or transmitter recovery and mortality cause determination (e.g., Panter et al. 2025). If a mortality was confirmed, we estimated date of death to be when total distance traveled failed to exceed GPS error (±20 m) over a 24‐h period. When feasible, mortality sources were determined by veterinary necropsy (e.g., infection, gunshot wounds) if a recovery was made within 5 days of final transmission, or inferred based on environmental context (e.g., electrocution if under a transformer/power line). In other cases where the last known location was accessible, successful recovery attempts led to only the transmitter being found due to detachment (i.e., harness failure), preventing vulture fate to be concluded.

Vultures marked with only patagial wing tags (hereafter “mark‐encounter vultures”) were incidentally encountered or recovered, and in turn reported, by personnel from wildlife management agencies (e.g., United States Department of Agriculture Wildlife Services) and community science participants via submissions to USGS Bird Banding Lab until the end of the study. We omitted reported sightings of transmitter vultures because re‐encounters preceded final transmission dates and were thus uninformative. Mortality causes for recovered mark‐encounter vultures were reported after lethal management actions (i.e., incidental take at conflict sites) or inferred based on environmental context (e.g., collision if along roadway) as post‐mortem changes typically destroyed or obscured vital diagnostic evidence. Since recoveries of mark‐encounter vultures largely relied on reports submitted by community science participants, unaware of the need to deduce mortality source, circumstantial evidence was not always provided. All incidental encounters and dead recoveries, as well as their mortality sources, were annotated in each monitoring dataset to the extent possible. After standardizing the satellite telemetry and incidental mark‐encounter data into a consistent format, we consolidated all data sources for downstream analysis.

### 2.5 Environmental Variables

Using publicly available Geographic Information System (GIS) data, we annotated location records of transmitter vultures with environmental variables representative of the transmitter study period, 2006–2025. We used human population density (people, km^−2^), primary and secondary road density (road, km km^−2^), and electrical power line density (line, km km^−2^) as metrics of human development. We obtained vector data of U.S. electric power lines from the “Homeland Infrastructure Foundation‐Level Data” (HIFLD 2023) dataset to generate raster files using the package “terra” (v. 1.8.42; Hijmans et al. 2022) in the R software environment (v. 4.5.0; R Core Team 2025). Here, we calculated the line density in each pixel using a search radius of 1000 m. We then obtained raster‐formatted human density and road density data from the “Gridded Population of the World” (Center For International Earth Science Information Network 2017) and “TIGER/Line Roads” (U.S. Census Bureau 2014) datasets, respectively, allowing us to extract geospatial values at a 1000‐m spatial resolution. For landscape composition and configuration metrics, we obtained raster‐formatted land cover data (500‐m grain size) from the “MODIS Terra MCD12Q1” dataset (Friedl and Sulla‐Menashe 2022). All rasters were aligned and resampled to 1000‐m spatial resolution. Landscape metrics were then calculated using the R package “landscapemetrics” (v. 2.2.1; Hesselbarth et al. 2019) with the 8‐neighbor rule and a circular moving window with a 2500‐m radius (19.6 km^2^). Following Turner and Gardner (2015), landscape composition metrics included the total number of land cover types (patch richness) and an index of land cover proportions of each type (Shannon diversity index), whereas configuration metrics included number of patches, patch density (number of patches 100 ha^−1^), largest patch index (percentage), and edge density (m ha^−1^).

### 2.6 Mortality Risk and Survival Analysis

To assess vulture mortality risk and survival, we built extended Cox proportional hazards models for the combined and transmitter‐only datasets using the R package ‘survival’ (v. 3.8.3; Therneau 2015). This regression model calculates hazard rates from a hazard function, which represents the instantaneous risk of an event occurring at a specific time point (Therneau 1997; Therneau and Grambsch 2000). From the model output, comparisons of hazard rates among two distinct levels of a predictor variable are provided as hazard ratios. Here, the cumulative sum of hazard rates (i.e., cumulative hazard) can be calculated and transformed into a survival function to estimate survival probabilities over time. For our analysis, we designated the starting time point as the day each vulture was released (i.e., entered at‐risk condition), while the event of interest was their verified mortality. We considered vultures alive from day of release until day of last re‐encounter or 1 day before transmitter data indicated no further movement. Vultures that were never recovered, or were alive when last located, were right‐censored.

Without discrete mark‐resight occasions standardized across release sites throughout the study (e.g., White and Burnham 1999), re‐encounter probability was unknown across space and time. Right‐censoring was thus largely susceptible to unknown heterogeneity in field efforts, which limited spatial delineation of sampling sites and temporal delineation of sampling occasions. Instead, the known time elapsed from first to last sighting among uncensored and right‐censored transmitter and mark‐encounter vultures was leveraged as a unifying piece of information (Heisey et al. 2007; Harju et al. 2020). To assess detection probability‐induced bias (Zhong and Hess 2009; Ranganathan and Pramesh 2012), we calculated the proportion of right‐censored vultures from the transmitter versus mark‐encounter datasets. A monthly follow‐up interval was used to track individual mortality events as conducted in Naveda‐Rodríguez et al. (2023), which for the additional analysis of transmitter data described later in this section, allowed us to pair a random location on the last day of each month and the first location on the day of mortality with their annotated environmental variables. To that end, this temporal resolution allowed us to circumvent data issues present at shorter intervals (e.g., weekly gaps in fixes) while accounting for variation in monthly home range fidelity and size (Holland et al. 2017; Morant et al. 2023).

Using the transmitter and combined datasets, we constructed two sets of survival models that included additive or interactive combinations of age class (HY, AHY, ASY) and season (breeding, non‐breeding) as predictor variables. To account for aging across monitoring periods, we transformed age class into a time‐varying covariate by assigning vultures that were alive after each successive year (365 days) with an older subgroup when applicable (e.g., HY to AHY). For vultures assigned an age class without certainty, particularly in discerning AHY from ASY, we delayed their entry time (365 days) to include them as the older age class. We also treated season as a time‐varying covariate by associating each monthly follow‐up with either the breeding or non‐breeding season. To account for latitudinal differences in breeding phenology, we defined the breeding season as February through July for vultures in northern states, and January through June for those in southern states (i.e., above versus below the 35th parallel north; Ferguson‐Lees and Christie 2001). Since black vultures are generally year‐long residents or short‐distance migrants in the northern extent of their range (Buckley et al. 2022), migratory seasons were not relevant here, and so all remaining months were defined as the non‐breeding season.

Using the environmental information from the transmitter dataset, we constructed an additional set of survival models that included additive combinations of human development metrics (human population, road, and electrical line density), landscape composition metrics (patch richness, Shannon diversity index), and landscape configuration metrics (number of patches, patch density, largest patch index, edge density) as predictor variables. All environmental variables were standardized prior to analysis and checked for multicollinearity using the variance inflation factor (VIF) metric in the R package “car” (v. 3.1.3; Fox et al. 2012). In doing so, we removed all highly correlated variables to improve estimate precision (VIF > 3; Zuur et al. 2010), including patch richness, edge density, and patch density.

To compare our fitted models, we calculated second order Akaike's Information Criterion (AICc) scores using the R package “AICcmodavg” (v. 2.3.2; Mazerolle 2023). While only considering models with low delta AICc scores (Δ_*i*_ < 4) and proportionate Akaike Weights (*W*_*i*_) for selection, we prioritized those more inclusive to interpreting the ecological basis of the survival response (Aho et al. 2014). To test the assumption of proportional hazard for each selected model (i.e., constant hazard ratio of predictor effects over time), we plotted parameter coefficients and visually inspected their slopes throughout the follow‐up period (slope approximating ‘0’ indicates proportionality). Chi‐square tests based on Schoenfeld residuals were also used to confirm no significant deviations from the proportional hazard assumption (*p* > 0.05; Grambsch and Therneau 1994). Model summary outputs provided us with estimates of log hazard ratio coefficients (*ß*) and their standard error (SE), hazard ratios (HR) and their 95% confidence intervals (95% CI), and *P* values for each predictor variable. Further, we calculated and reported annual survival point estimates and their 95% CI. Doing so allowed us to compare model estimates (absolute value differences) and precision (percent change in 95% CI spread) between the transmitter and combined datasets to evaluate efficacy and potential bias induced by the inclusion of mark‐encounter data. If a predictor variable was significant in the environmental model, we used the measured values to conduct model predictions of annual survival and cumulative hazard. We performed all data manipulation and visualization with the R packages: “dplyr” (v. 1.1.4; Wickham 2023), “broom” (v. 1.0.8; Robinson et al. 2025), “ggplot2” (v. 3.1.0; Wickham 2016), and “cowplot” (v. 0.9.3; Wilke 2015).

## 3 Results

The 28‐year monitoring period spanned from August 1997 to June 2025. Among the 184 transmitter vultures, 177 yielded analyzable data (i.e., no early harness or transmission failure). Of the 3281 marked vultures, 1353 were re‐encountered at least once after release, providing minimum survival times (Table 1). Marked vultures that were not re‐encountered (*n* = 1928) and transmitter vultures with failed deployments (*n* = 7) were not analyzed. The final dataset included 1530 individual vultures, wherein 174 were HY (11.4%), 552 were AHY (36.1%), and 804 were ASY (52.5%).

**TABLE 1:** Sample sizes for analyzing black vulture survival in the United States, 1997–2025.

Data source	*n*	Study period	Hatch year	After hatch year	After second year
Satellite Telemetry	177	2006–2025	20	52	105
Mark‐encounter	1353	1997–2025	154	500	699
Total	1530		174	552	804

Transmitter vultures were tracked for an average of 583 ± 40 days, whereas mark‐encounter vultures were re‐encountered over an average of 554 ± 14 days. The oldest vulture to be re‐encountered was at least 13 years old. Altogether, 1424 vultures were right‐censored and 106 were recovered. When considering detection probability‐induced bias, we had high rates of right‐censoring overall, but the proportion was slightly higher for mark‐encounter vultures (0.94) than transmitter vultures (0.85). The risk of bias was minimal for transmitter vultures since individuals could be actively relocated during each study period, that is unless a deployment ended prematurely (e.g., harness or transmission failure). For our mark‐encounter vultures, the potential risk was greater because they were less likely to be recovered after perishing from a natural source of mortality (e.g., succumbing to a fatal illness when out of public view).

### 3.1 Sources of Mortality

**FIGURE 3:**
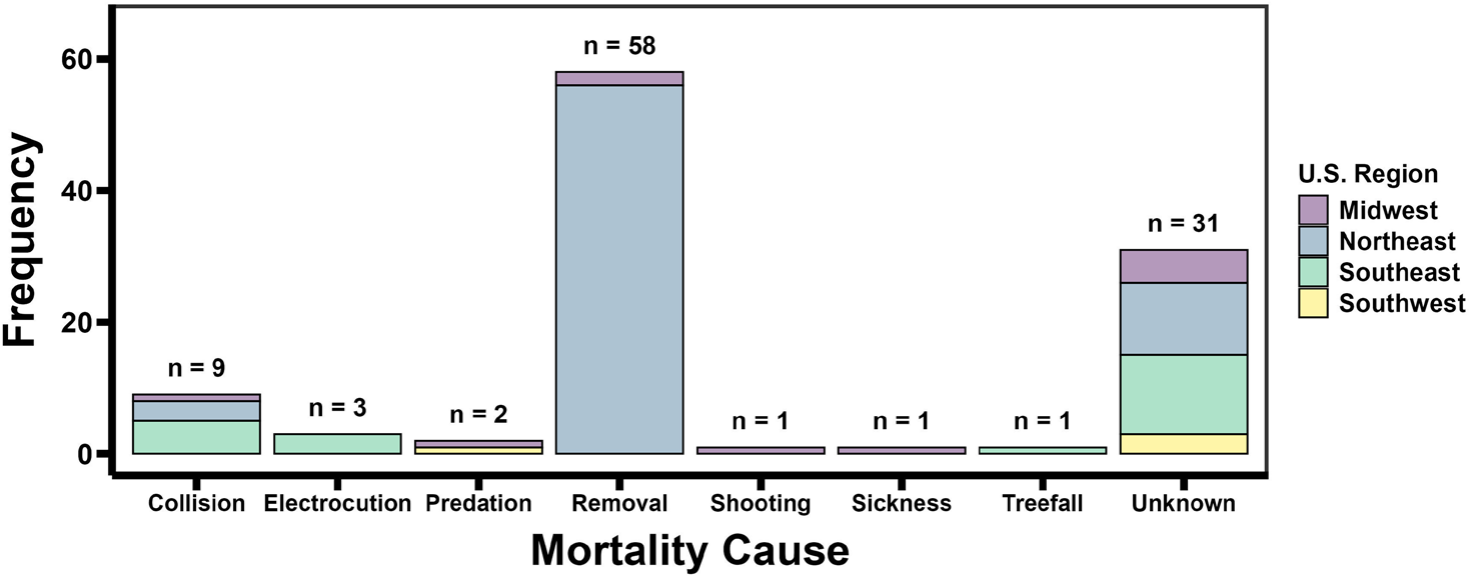
Frequencies for each source of mortality among recovered black vultures, 1997–2025.

Dead recoveries of tagged vultures (*n* = 106) allowed for an evaluation of mortality causes to the extent possible, wherein 67% were anthropogenic, 4% were natural, and the remaining 29% were unknown. Specifically, 55% were lethal removals, 8% were vehicle collisions, 3% were electrocutions, 1% was an illegal shooting, 2% were predation events, 1% was from sickness, and 1% was due to a treefall event (Figure 3). Among U.S. regions within the study area, mortalities largely occurred in the Northeast (66%), followed by the Southeast (20%), the Midwest (10%), and the Southwest (4%).

Lethal removals occurred at conflict sites in U.S. regions experiencing spatial expansion, that is, both the Northeast (97%) and the Midwest (3%). Vehicle collisions took place on primary roads across much of the study area, but most often occurred in the Southeast (56%), followed by the Northeast (33%), and the Midwest (11%). All known electrocutions occurred at transmission towers or power line transformers in the Southeast, while the only illegal shooting was in the Midwest. For known natural mortalities, predation events were evident in the Midwest (50%) and the Southwest (50%), while those owed to sickness (i.e., pneumonia) and natural disaster (i.e., treefall event) only occurred in the Midwest and the Southeast, respectively. Mortalities with unknown causes were discovered across the entire study area, with most in the Southeast (39%), followed by the Northeast (35%), the Midwest (16%), and the Southwest (10%).

**FIGURE 4:**
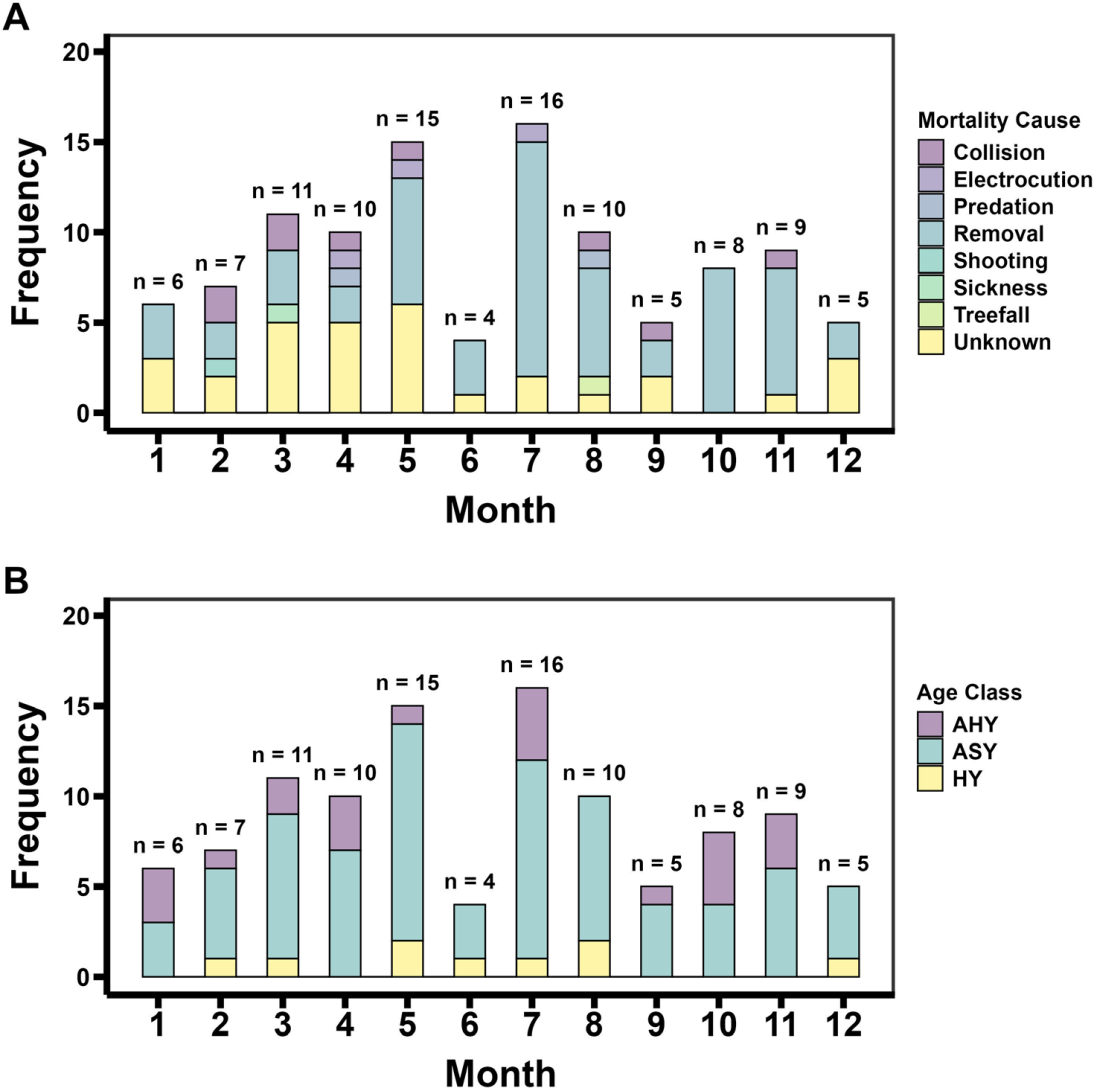
Monthly frequency of black vulture mortalities by (A) mortality cause and (B) age class (HY = Hatch Year, AHY = After Hatch Year, ASY = After Second Year).

Throughout the year, known mortalities occurred more often during the breeding season (60%) than the non‐breeding season (40%; Figure 4A). This pattern was largely driven by lethal removals, whereby 62% occurred during the breeding season and 38% in the non‐breeding season. Among age classes, most mortalities took place during the breeding season for ASY (64%) and HY (66%) vultures (Figure 4B), while AHY vultures had comparable mortality frequencies across the breeding (45%) and non‐breeding seasons (55%).

### 3.2 Survival Model Selection

The top‐ranked model for the combined dataset included age class and season as predictor variables (ΔAIC_c_ = 0.00, *w*_*i*_ = 0.61; Table 2). These parameter specifications also represented one of the plausible models for the transmitter dataset (ΔAIC_c_ = 3.48, *w*_*i*_ = 0.08), which we selected for estimate comparison. When we accounted for environmental variables exclusive to the transmitter dataset, the top‐ranked model included Shannon diversity index as a predictor variable (ΔAIC_c_ = 0.00; *w*_*i*_ = 0.52).

**TABLE 2:** Model selection results for black vulture survival analyses.

Survival models	*K*	AIC_c_	ΔAIC_c_	logLik	*w*_*i*_
**Combined dataset**					
**Age Class + Season**	**3**	**1349.77**	**0.00**	**−671.89**	**0.61**
Age Class * Season	5	1351.34	1.56	−670.67	0.28
Null	0	1359.79	10.02	−679.90	0.00
**Transmitter dataset (Precision analysis)**					
Null	0	251.51	0.00	−125.75	0.47
Season	1	252.29	0.79	−125.15	0.31
Age Class	2	254.14	2.63	−125.07	0.13
**Age Class + Season**	**3**	**254.99**	**3.48**	**−124.49**	**0.08**
**Transmitter dataset (Environmental analysis)**					
**Shannon diversity index**	**1**	**248.14**	**0.00**	**−123.07**	**0.52**
Edge density + Largest patch index	2	249.71	1.57	−122.85	0.24
Null	0	251.51	3.37	−125.75	0.11

*Note:* Only models with ∆AIC_c_ < 4 and null model are shown. Bolded rows indicate selected models.

### 3.3 Mortality Risk

**FIGURE 5:**
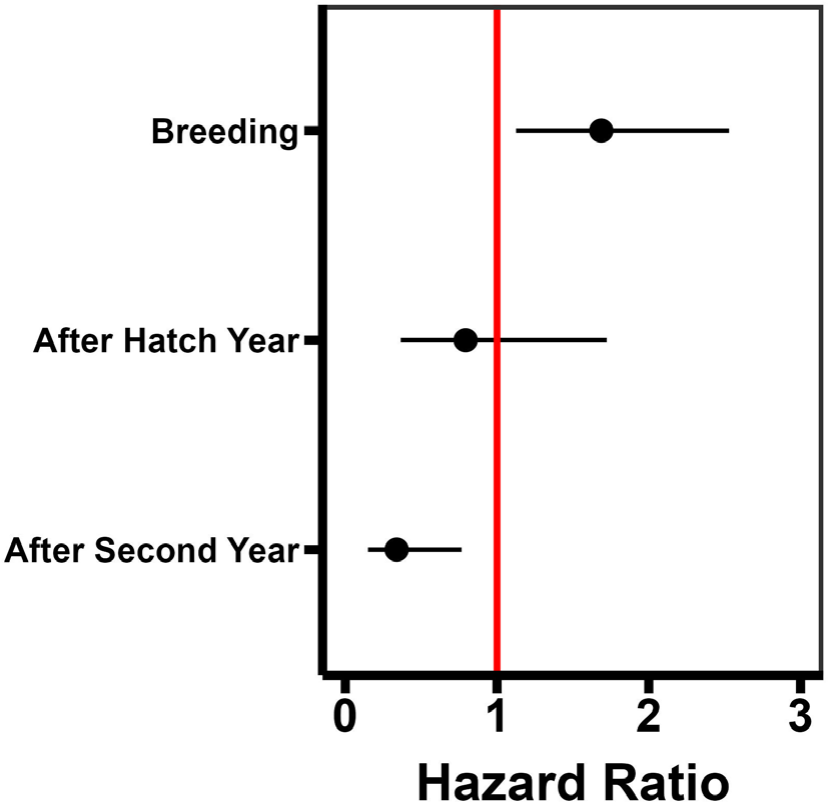
Hazard effect of season and age class for black vultures in the United States. Hazard ratios greater than one (red vertical line) indicate higher mortality risk and lower survival probability. Reference levels (hazard ratio = 1) include the non‐breeding season and vultures in their hatch year. Horizontal bars represent 95% CI of hazard ratio estimation.

**FIGURE 6:**
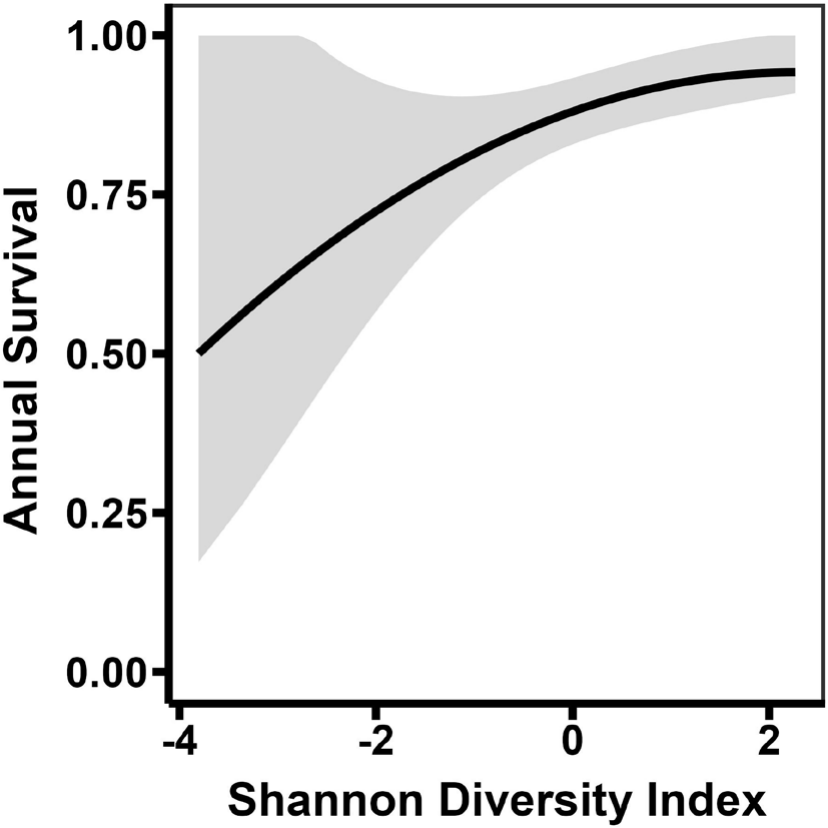
Predictive relationship between land cover diversity (Shannon diversity index) and annual survival probability for black vultures in the United States. Gray area represents 95% CI of the average relationship.

Throughout the year, mortality risk was significantly greater during the breeding season (*β* = 0.52, SE = 0.21, *p* = 0.011; Table 3, Figure 5), wherein vultures experienced 68.7% greater hazard relative to the non‐breeding season (HR = 1.69, 95% CI = 1.13–2.53). Among age classes, mortality risk was also significantly lower for older vultures (*β* = −1.08, SE = 0.42, *p* = 0.009), such that ASY vultures experienced 66.2% less hazard compared to HY vultures (HR = 0.34, 95% CI = 0.15–0.77). No effect in mortality risk was detected for AHY vultures (*β* = −0.23, SE = 0.40, *p* = 0.558) as their hazard overlapped both HY and ASY vultures (HR = 0.79, 95% CI = 0.37–1.72). In addition, mortality risk was significantly related to Shannon diversity index (*β* = −0.46, SE = 0.20, *p* = 0.019), whereby hazard decreased 36.7% for each unit increase in index value (HR = 0.63, 95% CI = 0.43–0.98). As such, higher index values were predictive of greater survival probability (Figure 6).

**TABLE 3:** Survival model summaries for black vultures.

Parameter	*β*	SE (*β*)	HR (95% CI)	Wald *Z*	*p*
Season|Age class model					
Breeding	0.52	0.21	1.69 (1.13–2.53)	2.53	0.011*
After hatch year	−0.23	0.40	0.79 (0.37–1.72)	−0.59	0.55
After second year	−1.08	0.42	0.34 (0.15–0.77)	−2.60	0.009*
Environmental model					
Shannon diversity index	−0.46	0.20	0.63 (0.43–0.93)	−2.35	0.019*

*Note:* Asterisk indicates significant parameter estimate (*p* < 0.05).

### 3.4 Annual Survival

Annual survival rates were high across age classes overall, averaging at 0.95 (95% CI: 0.92–0.98; Table 4). Among age classes, annual survival averaged 0.93 for HY vultures (0.88–0.98 95% CI, *n* = 174), 0.94 for AHY (0.92–0.97 95% CI, *n* = 552), and 0.97 for ASY vultures (0.96–0.99 95% CI, *n* = 804). Annual survival estimates from the transmitter dataset were consistently lower than those from the combined dataset, with absolute value differences in point estimates being ≤ 0.14 (e.g., the greatest difference was 0.79 vs. 0.93 for HY). Estimate precision for every age class was also markedly improved when analyzing the combined dataset, as the percentage decrease in 95% CI spreads was 74.3% for HY, 72.1% for AHY, and 84.2% for ASY (Table 4). Overall, the degree of improvement in annual survival estimates depended on the sample size of each age class in the transmitter‐only dataset.

**TABLE 4:** Annual survival estimates for black vultures in the United States, 1997–2025.

Age class	Annual survival		Spread of 95% CI		Precision increase
	Estimate	95% CI	Transmitter data	Combined data	
Hatch year	0.93	0.88, 0.98	0.38	0.10	74.3%
After hatch year	0.94	0.92, 0.97	0.19	0.05	72.1%
After second year	0.97	0.96, 0.99	0.17	0.03	84.2%

## 4 Discussion

By integrating multiple types of monitoring data into a time‐to‐event modeling framework, we addressed a long‐standing need for annual survival rates among black vultures of each age class in the United States. In doing so, we identified drivers of black vulture mortality and revealed seasonal and age‐related patterns in their mortality risk that would otherwise be unfeasible. Additionally, by deriving environmental information from satellite location data, we contributed to a growing body of evidence that vulture survival can be shaped by aspects of the landscape. Altogether, our findings support the hypotheses that survival in black vultures is comparable to other large‐sized raptors, and their mortality risk varies with age, season, and environmental conditions.

Dead recovery assessments revealed the largest mortality drivers to be anthropogenic (67% of cases) rather than natural (4% of cases), though these proportions may differ if our sample size was larger or the unknown causes (29% of cases) could be parsed. Most of the major mortality sources were evident across this species' range in the United States, and consistent with those identified in previous vulture studies around the globe (Ives et al. 2022). Notably, anthropogenic mortalities were similar in proportion for other vulture species in the United States (Kelly et al. 2015; Naveda‐Rodríguez et al. 2023). Such findings seemingly highlight the extent to which human activities can be generalized as a threat to vulture survival. However, lethal control across the study area, wherein vultures undeterred by non‐lethal management were removed from conflict sites, was a major factor among the anthropogenic mortalities. Due to how lethal removals are procedurally reported, the proportion of mortalities owed to this source is inherently biased (55% of cases), particularly when considering the limitations in recovering vultures that perished from other factors. Much of this bias may have been avoided if vultures were tagged randomly throughout the study area rather than where known human‐vulture conflicts occur. Yet, since removals of black vultures are a potential hazard like other known anthropogenic factors (e.g., electrocutions, collisions, illegal shootings; Ives et al. 2022), they were included to comprehensively account for all sources of mortality.

Interestingly, recoveries of lethal removals were entirely from management programs in the northern extent of their range (i.e., states in the Midwest and Northeast), including vultures tagged within the same state or from nearby states (e.g., Pennsylvania and West Virginia). A lack of lethally removed tagged vultures in southern states suggests that they were not encountered in conflict areas warranting lethal take, avoided management personnel during removal efforts, and/or experienced a lower incidence of lethal control overall. Unlike human‐vulture interactions in the southern United States, where conflicts are well known and mitigation strategies better established, those arising in recent expansion areas could be perceived as a greater threat, prompting the rise in requests for lethal control noted in Zimmerman et al. (2019). Considering this species' historical range and resulting latitudinal differences in abundance (i.e., more vultures in southern states; Zimmerman et al. 2019), the likelihood of removing tagged vultures may also be greater in northern states if they represent a greater proportion of the subpopulation. In any case, lethal removals of black vultures reported herein reflect the various outcomes of ongoing conflict management throughout the United States.

Annual survival estimates were nonetheless high overall (≥ 0.93), with data supplemented from mark‐encounter vultures increasing point estimate values and reducing uncertainty across age classes. As in previous studies, deriving estimates using this analytical approach seems beneficial when satellite telemetry sample sizes are relatively small, and a reasonable amount of mark‐encounter data are available (Harju et al. 2020; but see DeCesare et al. 2016). The annual survival rates reported herein are consistent with previous studies on black vultures of smaller spatial extents (≥ 0.88; Rabenold 1986; Rabenold and Decker 1990), other vultures inhabiting the United States (≥ 0.92; Kelly et al. 2015; Naveda‐Rodríguez et al. 2023), and large‐size raptors in general (> 0.9; Newton et al. 2016). The age‐related survival pattern we found is also akin to other raptor species, that is, lower survival in the first and pre‐breeding years of life (0.93 for HY and 0.94 for AHY) than in subsequent years (0.97 for ASY). High annual survival, especially among adults, is likely to help to explain this *K*‐selected species' population growth and range expansion (Stahl and Oli 2006). Yet, survival estimates for each age class should also better inform future models of allowable take, particularly when calculating intrinsic growth rates within a prescribed take level framework (Runge et al. 2009; Zimmerman et al. 2019).

Age class comparisons also revealed greater mortality risk for HY vultures than for ASY vultures (66.2% hazard increase), coinciding with demographic patterns often observed in other vulture species (e.g., Chantepie et al. 2016; Sanz‐Aguilar et al. 2017; Mallord et al. 2024). Following life history theory (Stearns 1992), the first year is likely most critical for survival as vultures must develop their flying, foraging, navigational, and social skills or perish (e.g., Gómez‐López et al. 2025). Conversely, older and more adept vultures are assumedly better established within their home ranges and less susceptible to mortality sources (e.g., starvation, predation; Hill et al. 2019; Ives et al. 2022). The intermediate AHY estimates may therefore represent the progression in overcoming ecological challenges, while the observed overlap HY and ASY estimates could be owed to the heterogeneity of actual vulture ages within this subgroup. In any case, our reported age class variation in mortality risk lends support to the emerging demographic patterns detected across raptor species (Newton et al. 2016).

Seasonal comparisons in mortality risk revealed greater hazard during the breeding season, particularly for ASY and HY vultures. With black vultures generally being year‐round residents (Buckley et al. 2022), devoid of the costs of long‐distance migration (Newton 2025), a seasonal pattern in mortality risk was unexpected. Instead, adult survival may become compromised due to activities linked to reproduction. Throughout this period, nesting pairs of black vultures frequently occupy man‐made structures (Buckley et al. 2022), which could prompt illegal killings by property owners or lethal removals from management programs. Greater foraging risks may also be required to meet the energetic demands of reproduction, exposing vultures to additional mortality factors (e.g., vehicle collisions) if residing in human‐modified landscapes. In addition, food sources may become more unpredictable or scarce throughout this period (e.g., limited carrion availability, faster carcass decomposition rates; Moleón et al. 2019). Such conditions are assumedly harsh for all age classes, but especially for developing juveniles as their mortalities increased until late‐breeding season. Granted lethal removals, the largest driver of mortality, occurred more often during the breeding (62% of cases) versus non‐breeding season (38% of cases), mortality risk was likely compounded by this factor among others coinciding with the breeding season. As such, management programs should take precautions when employing lethal control methods during this more sensitive period of the year (e.g., removing less).

Our finding that vulture mortality risk was lower in areas with greater land cover diversity (i.e., Shannon index values) is corroborated by the associated benefits, including stronger thermal currents for facilitating flight and greater resource availability (Novoselova et al. 2020, 2022). In certain situations where landscape transformations increase local diversity (e.g., early fragmentation of a large area), vultures can also benefit from complementation through increased obstruction currents for additional soaring opportunities and alternating habitats for foraging, roosting, and nesting (Thompson et al. 1990; Hartel et al. 2008). Yet, as landscapes become increasingly transformed and less diverse (e.g., urbanized land cover), the associated survival benefits presumably diminish, especially because black vultures generally select against such areas in studied portions of the United States (Hill et al. 2021).

Despite the expectation that human development would impact black vulture survival, our anthropogenic metrics were not associated with mortality risk across the landscape. Areas with greater human population density may not necessitate activities detrimental to vulture survival so long as local habitat is accommodating. Here, the benefits may outweigh the risks of being in proximity to humans (e.g., nearby food access; Novaes and Cintra 2013, 2015), as black vultures and other vulture species can persist in densely human populated areas (de Araujo et al. 2018; Henriques et al. 2018). Moreover, the corresponding aspects of human development considered here, electrical line and road density, were not directly implicated in survival either. Although we found electrocutions and vehicle collisions to be two of the larger sources of mortality, the density of these infrastructural components may not necessarily lead to greater risk. However, road density was found to be a significant hazard to vulture survival (Arrondo et al. 2020; Naveda‐Rodríguez et al. 2023), a relationship assumedly owed to being attracted to greater availabilities of road‐killed carcasses (Lambertucci et al. 2009). Considering most dead recoveries were from mark‐encounter vultures, which were excluded from the environmental models, the full impact of these factors may have been unaccounted for in our analysis. Additionally, the temporal and spatial resolution of our data may have further obscured effects on mortality risk if at all present (e.g., Evans et al. 2015). As such, the occurrence of these mortality factors should still be considered under vulture management programs.

Regarding vulture conservation and management, assessing survival is an essential yet challenging task that we have largely accomplished for black vultures monitored in the United States. Our time‐to‐event modeling approach allowed for survival estimates across a large portion of the U.S. range that black vultures currently inhabit, which should help ascertain effective and sustainable levels of allowable take. Relatively high survival rates did not indicate this species to be of conservation concern, but rather corroborated the reported population increases and range expansion. Yet, the identified seasonal and age‐related vulnerabilities should be considered during lethal control of human–vulture conflicts to better ensure population sustainability. Black vultures and other avian scavengers may also be at less risk if diverse landscapes are conserved where possible. Moving forward, additional aspects of black vulture demography should be investigated so that population management can be applied more effectively. Modeling approaches that integrate multiple data sources, such as those included herein, should be useful in better understanding the population dynamics of this species and others of concern.

## Author Contributions

**Spencer B. Hudson:** data curation (lead), formal analysis (lead), investigation (lead), methodology (lead), software (lead), validation (lead), visualization (lead), writing – original draft (lead), writing – review and editing (lead). **Eric A. Tillman:** data curation (lead), writing – review and editing (supporting). **Marian L. Wahl:** data curation (supporting). **Patrick A. Zollner:** funding acquisition (supporting), project administration (supporting), writing – review and editing (supporting). **Caryn D. Ross:** data curation (supporting), writing – review and editing (supporting). **Travis L. DeVault:** data curation (supporting), funding acquisition (supporting), project administration (supporting), writing – review and editing (supporting). **James C. Beasley:** data curation (supporting), funding acquisition (supporting), project administration (supporting), writing – review and editing (supporting). **Adrián Naveda‐Rodríguez:** data curation (supporting), writing – review and editing (supporting). **Scott A. Rush:** data curation (supporting), writing – review and editing (supporting). **Noah M. Osterhoudt:** data curation (supporting), writing – review and editing (supporting). **Jeffrey F. Kelly:** funding acquisition (supporting), project administration (supporting). **Adam E. Duerr:** data curation (supporting), writing – review and editing (supporting). **Lee A. Humberg:** funding acquisition (supporting), project administration (supporting). **Brett G. Dunlap:** investigation (supporting), project administration (supporting). **Chad Neil:** data curation (supporting), investigation (supporting). **John T. Forbes:** data curation (supporting), funding acquisition (supporting), project administration (supporting). **Harris Glass:** data curation (supporting), funding acquisition (supporting). **Travis L. Guerrant:** funding acquisition (equal), project administration (equal). **Robert W. Byrd:** funding acquisition (supporting), project administration (supporting). **Philip W. Kavouriaris:** data curation (supporting), writing – review and editing (supporting). **Andrea K. Darracq:** data curation (supporting), writing – review and editing (equal). **Matthew T. Springer:** data curation (supporting), writing – review and editing (supporting). **Bryan M. Kluever:** conceptualization (equal), funding acquisition (equal), project administration (equal), supervision (equal), writing – review and editing (equal).

## Funding

This work was supported by Animal and Plant Health Inspection Service (AP23WSNWRC00C103). Office of Environmental Management (DE‐EM0005228). College of Agriculture, Purdue University. National Wildlife Research Center.

## Conflicts of Interest

The authors declare no conflicts of interest.

## Data Availability

All data related to this article are archived in the Dryad Digital Repository (https://doi.org/10.5061/dryad.vx0k6dk69).

## References

[ece373226-bib-0001] Aho, K. , D. Derryberry , and T. Peterson . 2014. “Model Selection for Ecologists: The Worldviews of AIC and BIC.” Ecology 95: 631–636. 24804445 10.1890/13-1452.1

[ece373226-bib-0002] Arrondo, E. , A. Sanz‐Aguilar , J. M. Pérez‐García , A. Cortés‐Avizanda , J. A. Sánchez‐Zapata , and J. A. Donázar . 2020. “Landscape Anthropization Shapes the Survival of a Top Avian Scavenger.” Biodiversity and Conservation 29: 1411–1425.

[ece373226-bib-0098] Avery, M. L. 2004. “Trends in North American Vulture Populations.” Proceedings of the Vertebrate Pest Conference 21: 116–121.

[ece373226-bib-0004] Avery, M. L. , J. S. Humphrey , T. S. Daughtery , et al. 2011. “Vulture Flight Behavior and Implications for Aircraft Safety.” Journal of Wildlife Management 75: 1581–1587.

[ece373226-bib-0003] Avery, M. , and M. Lowney . 2016. Vultures. Wildlife Damage Management Technical Series. US Department of Agriculture, Animal and Plant Health Inspection Service, Wildlife Services, National Wildlife Research Center.

[ece373226-bib-0006] Blackwell, B. F. , M. L. Avery , B. D. Watts , and M. S. Lowney . 2007. “Demographics of Black Vultures in North Carolina.” Journal of Wildlife Management 71: 1976–1979.

[ece373226-bib-0007] Blackwell, B. F. , and S. E. Wright . 2006. “Collisions of Red‐Tailed Hawks ( *Buteo jamaicensis* ), Turkey Vultures ( *Cathartes aura* ), and Black Vultures ( *Coragyps atratus* ) With Aircraft: Implications for Bird Strike Reduction.” Journal of Raptor Research 40: 76–80.

[ece373226-bib-0009] Buckley, N. J. 1996. “Food Finding and the Influence of Information, Local Enhancement, and Communal Roosting on Foraging Success of North American Vultures.” Auk 113: 473–488.

[ece373226-bib-0008] Buckley, N. J. , B. M. Kluever , R. Driver , and S. A. Rush . 2022. “Black Vulture (*Coragyps atratus*), Version 2.0.” In Birds of the World. Cornell Lab of Ornithology. 10.2173/bow.blkvul.02.

[ece373226-bib-0010] Callaghan, C. T. , S. Nakagawa , and W. K. Cornwell . 2021. “Global Abundance Estimates for 9,700 Bird Species.” Proceedings of the National Academy of Sciences 118: e2023170118. 10.1073/pnas.2023170118 PMC8166167 34001610

[ece373226-bib-0011] Center For International Earth Science Information Network‐CIESIN‐Columbia University . 2017. Gridded Population of the World, Version 4 (GPWv4): Population Density, Revision 11 (Version 4.11). Palisades, NY Socioeconomic Data and Applications Center (SEDAC).

[ece373226-bib-0012] Chantepie, S. , C. Teplitsky , S. Pavard , et al. 2016. “Age‐Related Variation and Temporal Patterns in the Survival of a Long‐Lived Scavenger.” Oikos 125: 167–178.

[ece373226-bib-0013] Conover, M. R. , and D. O. Conover . 2022. Human‐Wildlife Interactions: From Conflict to Coexistence. CRC Press.

[ece373226-bib-0014] de Araujo, G. M. , C. A. Peres , F. B. Baccaro , and R. S. Guerta . 2018. “Urban Waste Disposal Explains the Distribution of Black Vultures (*Coragyps atratus*) in an Amazonian Metropolis: Management Implications for Birdstrikes and Urban Planning.” PeerJ 6: e5491. 30233993 10.7717/peerj.5491 PMC6140672

[ece373226-bib-0015] DeCesare, N. J. , M. Hebblewhite , P. M. Lukacs , and D. Hervieux . 2016. “Evaluating Sources of Censoring and Truncation in Telemetry‐Based Survival Data.” Journal of Wildlife Management 80: 138–148.

[ece373226-bib-0016] DeVault, T. L. , J. C. Beasley , Z. H. Olson , et al. 2016. “Ecosystem Services Provided by Avian Scavengers.” In Why Birds Matter: Avian Ecological Function and Ecosystem Services, 235–270. University of Chicago Press.

[ece373226-bib-0017] DeVault, T. L. , B. D. Reinhart , I. L. Brisbin Jr. , and O. E. Rhodes Jr. 2005. “Flight Behavior of Black and Turkey Vultures: Implications for Reducing Bird–Aircraft Collisions.” Journal of Wildlife Management 69: 601–608.

[ece373226-bib-0018] DeVault, T. L. , J. Rhodes , E. Olin , and J. A. Shivik . 2003. “Scavenging by Vertebrates: Behavioral, Ecological, and Evolutionary Perspectives on an Important Energy Transfer Pathway in Terrestrial Ecosystems.” Oikos 102: 225–234.

[ece373226-bib-0019] Evans, B. A. , J. S. Humphrey , E. A. Tillman , M. L. Avery , and B. M. Kluever . 2024. “Site‐Specific Space Use and Resource Selection by Black Vultures (*Coragyps atratus*) in the Southeastern USA.” Ibis 166: 129–145.

[ece373226-bib-0020] Evans, B. S. , T. B. Ryder , R. Reitsma , A. H. Hurlbert , and P. P. Marra . 2015. “Characterizing Avian Survival Along a Rural‐To‐Urban Land Use Gradient.” Ecology 96: 1631–1640.

[ece373226-bib-0021] Ferguson‐Lees, J. , and D. A. Christie . 2001. Raptors of the World. A&C Black.

[ece373226-bib-0022] Fox, J. , S. Weisberg , D. Adler , et al. 2012. Package ‘Car’. Vol. 16, 333. R Foundation for Statistical Computing.

[ece373226-bib-0023] Friedl, M. , and D. Sulla‐Menashe . 2022. MODIS/Terra+Aqua Land Cover Type Yearly L3 Global 500 m SIN Grid V061.

[ece373226-bib-0024] Gómez‐López, G. , F. Martínez , A. Sanz‐Aguilar , M. Carrete , and G. Blanco . 2025. “Long‐Term Monitoring Reveals Sex‐and Age‐Related Survival Patterns in Griffon Vultures.” Journal of Zoology 325: 49–60.

[ece373226-bib-0025] Grambsch, P. M. , and T. M. Therneau . 1994. “Proportional Hazards Tests and Diagnostics Based on Weighted Residuals.” Biometrika 81: 515–526.

[ece373226-bib-0026] Harju, S. M. , S. M. Cambrin , R. C. Averill‐Murray , M. Nafus , K. J. Field , and L. J. Allison . 2020. “Using Incidental Mark‐Encounter Data to Improve Survival Estimation.” Ecology and Evolution 10: 360–370. 31988732 10.1002/ece3.5900 PMC6972812

[ece373226-bib-0027] Hartel, T. R. , C. I. Moga , K. Öllerer , et al. 2008. “A Proposal Towards the Incorporation of Spatial Heterogeneity Into Animal Distribution Studies in Romanian Landscapes.” North‐Western Journal of Zoology 4: 173–188.

[ece373226-bib-0028] Heisey, D. M. , T. L. Shaffer , and G. C. White . 2007. “The ABCs of Nest Survival: Theory and Application From a Biostatistical Perspective.” Studies in Avian Biology 34: 5.

[ece373226-bib-0029] Henriques, M. , J. P. Granadeiro , H. Monteiro , et al. 2018. “Not in Wilderness: African Vulture Strongholds Remain in Areas With High Human Density.” PLoS One 13: e0190594. 29385172 10.1371/journal.pone.0190594 PMC5791984

[ece373226-bib-0030] Hesselbarth, M. H. , M. Sciaini , K. A. With , K. Wiegand , and J. Nowosad . 2019. “Landscapemetrics: An Open‐Source R Tool to Calculate Landscape Metrics.” Ecography 42: 1648–1657.

[ece373226-bib-0031] Hijmans, R. J. , R. Bivand , K. Forner , J. Ooms , E. Pebesma , and M. D. Sumner . 2022. Package ‘Terra’, 384. Maintainer.

[ece373226-bib-0032] Hill, J. E. , T. L. DeVault , and J. L. Belant . 2019. “Cause‐Specific Mortality of the World's Terrestrial Vertebrates.” Global Ecology and Biogeography 28: 680–689.

[ece373226-bib-0033] Hill, J. E. , K. F. Kellner , B. M. Kluever , et al. 2021. “Landscape Transformations Produce Favorable Roosting Conditions for Turkey Vultures and Black Vultures.” Scientific Reports 11: 14793. 34285264 10.1038/s41598-021-94045-3 PMC8292396

[ece373226-bib-0034] Holland, A. E. , M. E. Byrne , A. L. Bryan , T. L. DeVault , O. E. Rhodes , and J. C. Beasley . 2017. “Fine‐Scale Assessment of Home Ranges and Activity Patterns for Resident Black Vultures ( *Coragyps atratus* ) and Turkey Vultures ( *Cathartes aura* ).” PLoS One 12: e0179819. 28678813 10.1371/journal.pone.0179819 PMC5497974

[ece373226-bib-0035] Homeland Infrastructure Foundation‐Level Data (HIFLD) . 2023. Electric Power Transmission Lines.

[ece373226-bib-0036] Humphrey, J. S. , and M. L. Avery . 2014. “Improved Satellite Transmitter Harness Attachment Technique.” Journal of Raptor Research 48: 289–291.

[ece373226-bib-0037] Ives, A. M. , M. Brenn‐White , J. Y. Buckley , C. J. Kendall , S. Wilton , and S. L. Deem . 2022. “A Global Review of Causes of Morbidity and Mortality in Free‐Living Vultures.” EcoHealth 19: 40–54. 35000042 10.1007/s10393-021-01573-5

[ece373226-bib-0038] Kelly, T. R. , B. A. Rideout , J. Grantham , et al. 2015. “Two Decades of Cumulative Impacts to Survivorship of Endangered California Condors in California.” Biological Conservation 191: 391–399.

[ece373226-bib-0039] Kluever, B. M. , B. A. Evans , N. M. Osterhoudt , and E. A. Tillman . 2024. “Efficacy of an Inflatable Deterrent for Reducing New World Vulture Human‐Wildlife Conflict.” Scientific Reports 14: 6622. 38503812 10.1038/s41598-024-56941-2 PMC10951350

[ece373226-bib-0040] Kluever, B. M. , M. B. Pfeiffer , S. C. Barras , B. G. Dunlap , and L. A. Humberg . 2020. “Black Vulture Conflict and Management in the United States.” Human‐Wildlife Interactions 14: 376–389.

[ece373226-bib-0041] Lambertucci, S. A. , K. L. Speziale , T. E. Rogers , and J. M. Morales . 2009. “How Do Roads Affect the Habitat Use of an Assemblage of Scavenging Raptors?” Biodiversity and Conservation 18: 2063–2074.

[ece373226-bib-0042] Mallord, J. W. , K. P. Bhusal , A. B. Joshi , et al. 2024. “Survival Rates of Wild and Released White‐Rumped Vultures (*Gyps bengalensis*), and Their Implications for Conservation of Vultures in Nepal.” Ibis 166: 971–985.

[ece373226-bib-0043] Mazerolle, M. 2023. AICcmodavg: Model Selection and Multimodel Inference Based on (Q) AIC (c). R Package Version 2.3–2, 2023.

[ece373226-bib-0044] McClure, C. J. , L. Dunn , E. R. Buechley , et al. 2022. “Conservation Assessment of Raptors Within the USA and Canada.” Biological Conservation 272: 109633.

[ece373226-bib-0045] McClure, C. J. , and B. W. Rolek . 2020. “Relative Conservation Status of Bird Orders With Special Attention to Raptors.” Frontiers in Ecology and Evolution 8: 593941.

[ece373226-bib-0046] McClure, C. J. , J. R. Westrip , J. A. Johnson , et al. 2018. “State of the World's Raptors: Distributions, Threats, and Conservation Recommendations.” Biological Conservation 227: 390–402.

[ece373226-bib-0048] Moleón, M. , N. Selva , M. M. Quaggiotto , D. M. Bailey , A. Cortés‐Avizanda , and T. L. DeVault . 2019. “Carrion Availability in Space and Time.” In Carrion Ecology and Management, 23–44. Springer International.

[ece373226-bib-0049] Morant, J. , E. Arrondo , J. A. Sánchez‐Zapata , et al. 2023. “Large‐Scale Movement Patterns in a Social Vulture Are Influenced by Seasonality, Sex, and Breeding Region.” Ecology and Evolution 13: e9817. 36789342 10.1002/ece3.9817 PMC9909000

[ece373226-bib-0050] Naveda‐Rodríguez, A. , K. L. Bildstein , D. R. Barber , et al. 2023. “Turkey Vulture Survival is Reduced in Areas of Greater Road Density.” Ornithological Applications 125: duad024.

[ece373226-bib-0051] Newton, I. 2025. “Migration Mortality in Birds.” Ibis 167: 106–123.

[ece373226-bib-0052] Newton, I. , M. J. McGrady , and M. K. Oli . 2016. “A Review of Survival Estimates for Raptors and Owls.” Ibis 158: 227–248.

[ece373226-bib-0053] Novaes, W. G. , and R. Cintra . 2013. “Factors Influencing the Selection of Communal Roost Sites by the Black Vulture *Coragyps atratus* (Aves: Cathartidae) in an Urban Area in Central Amazon.” Zoologia (Curitiba) 30: 607–614.

[ece373226-bib-0054] Novaes, W. G. , and R. Cintra . 2015. “Anthropogenic Features Influencing Occurrence of Black Vultures ( *Coragyps atratus* ) and Turkey Vultures ( *Cathartes aura* ) in an Urban Area in Central Amazonian Brazil.” Condor: Ornithological Applications 117: 650–659.

[ece373226-bib-0055] Novoselova, N. S. , A. A. Novoselov , A. Macarrão , G. Gallo‐Ortiz , and W. R. Silva . 2020. “Remote Sensing Applications for Abating Aircraft‐Bird Strike Risks in Southeast Brazil.” Human‐Wildlife Interactions 14: 25–42.

[ece373226-bib-0056] Novoselova, N. S. , A. A. Novoselov , A. Macarrão , G. Gallo‐Ortiz , and W. R. Silva . 2022. “Thermal Circulation Affects Black Vulture *Coragyps atratus* Soaring Behaviour in the Vicinity of Two Airports in South‐East Brazil.” Vulture News 82: 1–13.

[ece373226-bib-0057] Ogada, D. L. , F. Keesing , and M. Z. Virani . 2012. “Dropping Dead: Causes and Consequences of Vulture Population Declines Worldwide.” Annals of the New York Academy of Sciences 1249: 57–71. 22175274 10.1111/j.1749-6632.2011.06293.x

[ece373226-bib-0058] Omernik, J. M. , and G. E. Griffith . 2014. “Ecoregions of the Conterminous United States: Evolution of a Hierarchical Spatial Framework.” Environmental Management 54: 1249–1266. 25223620 10.1007/s00267-014-0364-1

[ece373226-bib-0059] Panter, C. T. , C. Nebel , M. Raab , et al. 2025. “A LEAP Forward in Wildlife Conservation: A Standardized Framework to Determine Mortality Causes in Large GPS‐Tagged Birds.” Ecology and Evolution 15, no. 4: e70975. 40170813 10.1002/ece3.70975 PMC11949540

[ece373226-bib-0060] Quinby, B. M. , B. M. Kluever , G. N. Burcham , et al. 2022. “Spatial Risk Modeling of Cattle Depredation by Black Vultures in the Midwestern United States.” Journal of Wildlife Management 86: e22231.

[ece373226-bib-0099] R Core Team . 2025. “R: A Language and Environment for Statistical Computing.” R Foundation for Statistical Computing. https://www.R‐project.org/.

[ece373226-bib-0062] Rabenold, P. P. 1986. “Family Associations in Communally Roosting Black Vultures.” Auk 103: 32–41.

[ece373226-bib-0061] Rabenold, P. , and M. Decker . 1990. Black Vultures in North Carolina: Statewide Population Surveys and Analysis of Chatham County Population Trends. Final Report, North Carolina Wildlife Resources Commission, Nongame and Endangered Wildlife Program, Contract 1989SG07.

[ece373226-bib-0063] Ranganathan, P. , and C. Pramesh . 2012. “Censoring in Survival Analysis: Potential for Bias.” Perspectives in Clinical Research 3: 40. 22347702 10.4103/2229-3485.92307 PMC3275994

[ece373226-bib-0064] Reznick, D. , M. J. Bryant , and F. Bashey . 2002. “R‐and K‐Selection Revisited: The Role of Population Regulation in Life‐History Evolution.” Ecology 83: 1509–1520.

[ece373226-bib-0065] Robinson, D. , A. Hayes , and S. Couch . 2025. Broom: Convert Statistical Objects Into Tidy Tibbles. R Package Version1.0.8.

[ece373226-bib-0066] Robinson, R. A. , C. M. Meier , W. Witvliet , M. Kéry , and M. Schaub . 2020. “Survival Varies Seasonally in a Migratory Bird: Linkages Between Breeding and Non‐Breeding Periods.” Journal of Animal Ecology 89: 2111–2121. 32383289 10.1111/1365-2656.13250

[ece373226-bib-0067] Runge, M. C. , J. R. Sauer , M. L. Avery , B. F. Blackwell , and M. D. Koneff . 2009. “Assessing Allowable Take of Migratory Birds.” Journal of Wildlife Management 73: 556–565.

[ece373226-bib-0068] Rush, S. A. , N. J. Buckley , P. A. Zollner , et al. 2023. “Science‐Driven Guidelines Needed to Better Manage and Conserve Black Vultures in North America.” Wildlife Letters 1: 79–82.

[ece373226-bib-0069] Rushing, C. S. , J. A. Royle , D. J. Ziolkowski Jr. , and K. L. Pardieck . 2020. “Migratory Behavior and Winter Geography Drive Differential Range Shifts of Eastern Birds in Response to Recent Climate Change.” Proceedings of the National Academy of Sciences 117: 12897–12903. 10.1073/pnas.2000299117 PMC7293646 32457137

[ece373226-bib-0070] Sæther, B.‐E. , and Ø. Bakke . 2000. “Avian Life History Variation and Contribution of Demographic Traits to the Population Growth Rate.” Ecology 81: 642–653.

[ece373226-bib-0071] Sanz‐Aguilar, A. , A. Cortés‐Avizanda , D. Serrano , et al. 2017. “Sex‐and Age‐Dependent Patterns of Survival and Breeding Success in a Long‐Lived Endangered Avian Scavenger.” Scientific Reports 7: 40204. 28074860 10.1038/srep40204 PMC5225485

[ece373226-bib-0072] Sarrazin, F. , C. Bagnolini , J. L. Pinna , E. Danchin , and J. Clobert . 1994. “High Survival Estimates of Griffon Vultures (*Gyps Fulvus fulvus*) in a Reintroduced Population.” Auk 111: 853–862.

[ece373226-bib-0073] Sauer, J. R. , K. L. Pardieck , D. J. Ziolkowski Jr. , et al. 2017. “The First 50 Years of the North American Breeding Bird Survey.” Condor: Ornithological Applications 119: 576–593.

[ece373226-bib-0074] Scholer, M. N. , M. Strimas‐Mackey , and J. E. Jankowski . 2020. “A Meta‐Analysis of Global Avian Survival Across Species and Latitude.” Ecology Letters 23: 1537–1549. 32696563 10.1111/ele.13573

[ece373226-bib-0075] Sergio, F. , G. Tavecchia , J. Blas , L. López , A. Tanferna , and F. Hiraldo . 2011. “Variation in Age‐Structured Vital Rates of a Long‐Lived Raptor: Implications for Population Growth.” Basic and Applied Ecology 12: 107–115.

[ece373226-bib-0076] Sibly, R. M. , and J. Hone . 2002. “Population Growth Rate and Its Determinants: An Overview.” Philosophical Transactions of the Royal Society of London. Series B, Biological Sciences 357: 1153–1170. 12396508 10.1098/rstb.2002.1117 PMC1693026

[ece373226-bib-0077] Stahl, J. T. , and M. K. Oli . 2006. “Relative Importance of Avian Life‐History Variables to Population Growth Rate.” Ecological Modelling 198: 23–39.

[ece373226-bib-0078] Stearns, S. C. 1992. The Evolution of Life Histories. Oxford University Press.

[ece373226-bib-0079] Sweeney, T. M. , J. D. Fraser , and J. S. Coleman . 1985. “Further Evaluation of Marking Methods for Black and Turkey Vultures.” Journal of Field Ornithology 56: 251–257.

[ece373226-bib-0081] Therneau, T. 2015. A Package for Survival Analysis in R. R Package Version, 2, 2014.

[ece373226-bib-0082] Therneau, T. M. 1997. “Extending the Cox Model.” In Proceedings of the First Seattle Symposium in Biostatistics: Survival Analysis, 51–84. Springer.

[ece373226-bib-0083] Therneau, T. M. , and P. M. Grambsch . 2000. “The Cox Model.” In Modeling Survival Data: Extending the Cox Model, 39–77. Springer.

[ece373226-bib-0084] Thompson, W. L. , R. H. Yahner , and G. L. Storm . 1990. “Winter Use and Habitat Characteristics of Vulture Communal Roosts.” Journal of Wildlife Management 54: 77–83.

[ece373226-bib-0085] Turner, M. G. , and R. H. Gardner . 2015. “Landscape Metrics.” In Landscape Ecology in Theory and Practice: Pattern and Process, 97–142. Springer.

[ece373226-bib-0086] U.S. Census Bureau . 2014. TIGER/Line Shapefiles: Roads. U.S. Department of Commerce.

[ece373226-bib-0087] Wahl, M. L. , G. N. Burcham , A. M. Herbert , et al. 2024. “Taphonomic Signatures of Early Scavenging by Black and Turkey Vultures.” PLoS One 19: e0307610. 39141675 10.1371/journal.pone.0307610 PMC11324135

[ece373226-bib-0088] Wahl, M. L. , B. McWherter , P. A. Zollner , et al. 2023. “Livestock Producers' Perceptions of the American Black Vulture Conflict in the Midwestern United States.” Wildlife Society Bulletin 47: e1440.

[ece373226-bib-0089] White, G. C. , and K. P. Burnham . 1999. “Program MARK: Survival Estimation From Populations of Marked Animals.” Bird Study 46: S120–S139.

[ece373226-bib-0090] Wickham, H. 2016. “Data Analysis.” In ggplot2: Elegant Graphics for Data Analysis, 189–201. Springer.

[ece373226-bib-0091] Wickham, H. 2023. dplyr: A Grammar of Data Manipulation. R Package Version 1.1.4, 2023.

[ece373226-bib-0092] Wilke, C. O. 2015. cowplot: Streamlined Plot Theme and Plot Annotations for’ggplot2’. CRAN: Contributed Packages.

[ece373226-bib-0093] Zhong, M. , and K. R. Hess . 2009. Mean Survival Time From Right Censored Data, 66. Collection of Biostatistics Research Archive. The Berkeley Electronic Press.

[ece373226-bib-0094] Zimmerman, G. S. , B. A. Millsap , M. L. Avery , J. R. Sauer , M. C. Runge , and K. D. Richkus . 2019. “Allowable Take of Black Vultures in the Eastern United States.” Journal of Wildlife Management 83: 272–282.

[ece373226-bib-0095] Zuur, A. F. , E. N. Ieno , and C. S. Elphick . 2010. “A Protocol for Data Exploration to Avoid Common Statistical Problems.” Methods in Ecology and Evolution 1: 3–14.

